# Qualitative developmental research among low income African American adults to inform a social marketing campaign for walking

**DOI:** 10.1186/1479-5868-10-33

**Published:** 2013-03-05

**Authors:** Dawn K Wilson, Sara M St George, Nevelyn N Trumpeter, Sandra M Coulon, Sarah F Griffin, Abe Wandersman, Melinda Forthofer, Barney Gadson, Porschia V Brown

**Affiliations:** 1 Department of Psychology, University of South Carolina, Columbia, SC, 29208, USA; 2 Department of Public Health Sciences, College of Heath, Education, and Human Development, Clemson University, Clemson, SC, 29634, USA; 3 Biostatistics and Epidemiology, Arnold School of Public Health, University of South Carolina, Columbia, SC, 29208, USA; 4 M.H. Newton Family Life Enrichment Center, Sumter, SC, USA

**Keywords:** Social marketing, African American, Walking, Physical activity, Health promotion

## Abstract

**Background:**

This study describes the development of a social marketing campaign for increasing walking in a low income, high crime community as part of the Positive Action for Today’s Health (PATH) trial.

**Methods:**

Focus groups were conducted with 52 African American adults (ages 18 to 65 yrs), from two underserved communities to develop themes for a social marketing campaign to promote walking. Participants responded to questions concerning social marketing principles related to product, price, place, promotion, and positioning for increasing neighbourhood walking.

**Results:**

Focus group data informed the development of the campaign objectives that were derived from the “5 Ps” to promote physical and mental health, social connectedness, safety, and confidence in walking regularly. Focus group themes indicated that physical and mental health benefits of walking were important motivators. Walking for social reasons was also important for overcoming barriers to walking. Police support from trusted officers while walking was also essential to promoting safety for walking. Print materials were developed by the steering committee, with a 12-month calendar and door hangers delivered to residents’ homes to invite them to walk. Pride Stride walks empowered community walkers to serve as peer leaders for special walking events to engage new walkers.

**Conclusions:**

Essential elements for developing culturally tailored social marketing interventions for promoting walking in underserved communities are outlined for future researchers.

## Background

National studies have shown that physical inactivity is more prevalent among African American adults than non-minority adults [1]. These findings have led to an increasing national priority for increasing knowledge about the determinants and mediating factors of physical inactivity, especially among minority communities [2-5]. Previous research has demonstrated that underserved communities (low-income, predominately minority) have a number of environmental barriers that limit their engagement in walking, including concerns about safety, stray dogs, lack of group participation, and lack of available facilities and opportunities for physical activity (PA) [6-16]. This study describes the formative development of a social marketing campaign that targeted improving perceptions of safety, access, psychosocial and social environmental barriers related to walking among residents living in low-income, high crime communities as part of the Positive Action for Today’s Health (PATH) trial [17].

Social marketing is an approach which targets specific audiences with marketing strategies to improve personal health and quality of life, such as increasing walking [18]. In general, there are five major components of developing social marketing strategies, which include the “5 Ps:” (1) product, (2) price, (3) place, (4) promotion, and (5) positioning. *Product* refers to the target behavior, such as increasing walking. *Price* refers to the social, economic, and psychological costs involved in adopting the behavior such as walking. *Place* refers to the setting, community context, or distribution channels for the product (walking behavior). *Promotion* includes all actions designed to make the audience aware of the ideas, behaviors, and benefits of walking that may include interpersonal communication, media messages, grassroots approaches, special events and incentives. *Positioning (also known as exchange),* refers to the framing of the product (walking behavior) so that the perceived benefits are maximized and the perceived costs are minimized. Additional principles of social marketing include an understanding of (1) audience segmentation, including the creation of tailored messages, incentives, and opportunities that appeal to community subgroups, (2) channel analysis, which entails identification of communication pathways most likely to reach identified community subgroups, (3) identifying target markets through consumer research and (4) process tracking for documenting adherence to planned strategies [18-22].

Social marketing is a widely accepted approach in the field of health promotion [23,24]. Research on social marketing and the promotion of walking and physical activity has been reviewed by Alcalay and Bell [20]. Previous investigators have typically failed to have measurable objective outcomes, apply behavioral theory, use audience segmentation beyond basic demographics, conduct consumer research about the audiences of interest, and pre-test concepts and health communication messages. Whereas other social marketing approaches for increasing PA have placed greater emphasis on the individual-level [20], the present social marketing campaign also highlights the social and community-wide benefits of walking. This study specifically expands on past qualitative work by using principles based on Social Ecological Theory [25,26] and Social Cognitive Theory [27,28] to explore social environmental barriers and facilitators for improving motivation and self-confidence for walking and exercising regularly. Compared to studies reviewed by Alcalay and Bell [20], those reviewed by Yancey and colleagues [29], which targeted ethnic minority communities, were more heavily focused on community norms and activities. The current social marketing campaign specifically targeted community norms by focusing on family and community connectedness in the overall approach. In addition, the PATH trial addressed previous limitations by using a formative evaluation process that incorporated behavioral principles, audience segmentation, and consumer audience input to create a social marketing strategy to promote community walking.

A growing body of evidence suggests it is important to tailor social marketing approaches to increase social environmental supports for walking. Previous research has focused on improving key social environmental factors including community connectedness, collective efficacy, social networks, and social support in developing social marketing approaches [30-40]. Specifically, previous investigators have examined these factors to more fully understand the relation between social factors and walking and PA outcomes [30-38,40]. In a review of interventions for promoting walking, it has demonstrated that social support was strongly and positively associated with increased levels of walking and PA [30,32,35,37,39,41]. In a study by McNeil et al. [36] both social and physical environmental supports had indirect effects on PA, and social support influenced PA through increasing motivation. Fisher et al. [33] found that community connectedness was significantly associated with increased levels of neighborhood walking and PA and positive social interactions for PA in adults. The social marketing approach in this study targeted intrapersonal level variables such as beliefs, motivation, and self-efficacy related to walking as well as social environmental factors such as perceptions of neighborhood safety, social support, and community connectedness.

The overall goal of the PATH trial was to develop and evaluate the efficacy and cost-effectiveness of a walking intervention for increasing PA in low-income, high crime communities [17]. Three communities were randomized to receive one of three programs: a police- patrolled walking program plus social marketing campaign (full intervention), a police-patrolled walking only program (walking only intervention), or no walking, general health education program (comparison program). This article describes the formative work to develop a social marketing intervention that targeted determinants of walking, neighborhood safety and access for walking, as well as psychological and social factors related to increasing walking in the full intervention community in South Carolina (SC) that specifically targeted older African American women and men.

## Methods

### PATH intervention community – focus groups

#### Participants

Post-randomization of the PATH trial [17], five focus groups were conducted in two underserved communities (including the PATH intervention community) to tailor the social marketing campaign to the specific needs of the community. Participants for the focus groups were recruited through the local neighbourhood association in Sumter, SC and through a local school that was in a neighbourhood that was matched on geographical demographics to Sumter including percent minorities, poverty, and crime rates. Participants (n = 52; mean age = 64 years) were primarily African American and included equal numbers of men and women (see Table 1). Community partners provided a list of contacts from local neighborhood associations and asked school staff who were older African American adults to participate given that the PATH trial would target a similar population for the walking program. All individuals on the list were contacted by telephone and personally invited to participate. Participants answered questions about walking in their community, including what they would want in a neighborhood walking program and how to overcome obstacles such as crime.

**Table 1:** Participant demographics

**Demographic characteristics**	**(n = 52)**
Gender	
Male	31.0
Female	69.0
Race	
African American	96.0
Caucasian	4.0
Age, years	
18-24	2.0
25-44	31.0
45-64	50.0
65+	12.0
Unknown	5.0
Marital Status	
Single	19.0
Married	60.0
Divorced	14.0
Separated	4.0
Widowed	0
Unknown	3.0

Note: the values are expressed as percentages.

#### Focus group protocols

The focus group sessions were conducted by the principal investigator and research staff using a standardized protocol of questions and probes. The protocol of questions and probes were pilot tested on African American staff at the University of South Carolina prior to conducting the focus groups. Participants completed an informed consent prior to participating and then a short demographic survey.

#### Data coding and analyses

The coding scheme was composed of “levels” of categorization of the qualitative data. An initial code book was developed based on expected responses and outlined theoretical constructs. After an initial review of the focus group transcripts, the code book was revised to more appropriately represent the data. As perceptions, ideas, and suggestions were identified, they were classified into categories or “themes,” which were ultimately used to summarize the data [42]. Decision rules were developed to guide and facilitate the coding of the data by using hypothetical situations to illustrate how responses were to be coded. Coders were provided a copy of both the code book and decision rules.

After the recorded focus groups were transcribed by an outside agency, the transcript was checked for accuracy against the original recordings. The transcript was coded by independent raters and inter-rater reliability estimates were calculated (*r* = .70). Individual raters met to discuss each coding disagreement until a consensus was met regarding the final codes. Once the transcript was coded the codes were entered into QSR NVivo for content analysis structures as outlined by Miles and Huberman [43]. Themes were defined as concepts discussed by at least two different participants. Codes were subsequently cross-referenced to each respective participant. Once codes were applied to all transcripts, QSR NVivo was used to extract coded participant responses. Separate analyses were conducted for men and women to assure that themes for both sexes could be integrated into the social marketing campaign.

## Results

### Focus groups

#### Product/Place themes for walking and PA

To determine how and where a program should be implemented, it was imperative to understand walking behavior and influences. Both men and women indicated that their neighborhood and local parks were their primary walking locations (see Table 2). Regarding neighborhood walking, one male participant said, “In my neighbourhood, and it’s just like I said, a nice circular, I call it a circuit that I can walk, nice hills and everything.” Males and females also had similar responses regarding walking partners. Both men and women reported walking with family members, including adults and children.

**Table 2:** Summary of results (Product/Place)

**Question & theme**	**Males**	**Females**
**Where participants walk**		
Neighborhood	“In my neighborhood, and it’s just like I said a nice circular, I call it a circuit that I can walk, nice hills and everything.”	“enjoying walking through the neighborhood”
Park	“We’d go walking together at Caughman Road Park.”	“there is and, it’s not in my neighborhood but it’s like three minutes away I can go to Caughman Road [Park] and walk”
**Who participants walk with**		
Adult family member	“I walk with my wife.”	“I walk with my spouse or sister-in-law.”
Child family member	“I go walking with my daughter, and my little son.”	“In the afternoon, me and my children usually walk”
Unspecified family member	---	“walk with…my family.”
Friends	---	“when I’m walking it will either be with friends or my children.”
Self	---	“I love to walk alone”
**What participants want in a walking trail**		
Trusted police officers	“I don’t care how well you feel and unless you got a police every 10 to 15 minutes. There’s some crazy people in this world.”	“we could have, you know, protection, police officer or whatever to ride around. You know, on a regular basis, you know. ”
Open view	“…if you’re walking you want to see your children.”	“because you know you want to make sure that somebody’s see you.”
Stations on trail	“…because if you have walking trails you have other things where the kids can play basketball or whatever, you know, the family will enjoy it because the adults may enjoy walking, but the kids not gonna walk.”	
Cleanliness/Aesthetic	“You can’t, you know, just can’t put it any place, you need to have it in a pleasant location, a pleasant site where it will be enjoyable for yourself and the kids.”	“Attract, attractiveness, you know, as you say, making it attractive where a, a place where people would want to be.”
Safety	“I like to walk on a path that’s safe, but I think you have to take time to look at, to look at the area and people that are gonna be using it because I think if you’re uncomfortable on it, be it male or female, you’re not gonna go on it.”	“More people would participate I think if they knew they had protection.”
Weather provisions	“When it’s summertime, we want to be outside. You know, we want to see the sun, we want to-all that stuff, flowers…when it’s cold we want to be inside not on that walking trail.”	“Would there be some provisions for inclement weather? If you want to, you know, if you’re a preacher of routine and you walk everyday at 5:00”
Signs		“You know those signs where you’re at this point and you’re, you can get to this point?”

When asked about desired aspects of a walking trail, participants provided a variety of responses. Males and females both reported wanting trusted police officers. For example, one male shared, “I don’t care how well you feel unless you got a police every 10 to 15 minutes…. There’s some crazy people in this world….” An open view of surroundings was also desired, “if you’re walking you want to see your children.” Participants also articulated the importance of trail cleanliness and attractiveness. “You can’t, you know, just can’t put it any place, you need to have it in a pleasant location, a pleasant site where it will be enjoyable for yourself and the kids.” Participants even went as far as thinking about how weather would affect the trail, with one participant asking, “Would there be some provisions for inclement weather?”

Safety was the most salient theme that emerged from data in the product portion of the formative focus groups. For example, “I like to walk on the path [that] can be set up that’s safe, but I think you have to take time to look at, to look at the area and people that are gonna be using it because I think if you’re uncomfortable on it, be it male or female, you’re not gonna go on it,“ and ”More people would participate I think if they knew they had protection.”

#### Product benefits for walking and PA

Although benefits of exercise and walking are commonly known, it was important to understand the participants’ perceptions of these benefits (see Table 3). Participants were first asked to share their opinions on the best things about exercise. Both males and females cited stress relief, and male participants additionally mentioned physical fitness and mental wellness as benefits. In addition to stress relief, female participants commented on increased energy, health, and weight-related benefits.

**Table 3:** Summary of results (Product Benefits)

**Question & theme**	**Males**	**Females**
**Best things about Exercising**		
Relieves stress/ tension	“I really enjoy working out because it’s a stress release.”	“enjoying walking through the neighborhood”
Being physically fit	“it gives me a joy to, to be able to exercise my body so that I can stay physically fit.”	---
Mental wellness	“it helps you clear your mind to just feel better, especially when you do it in the morning times, you know. You just feel better all day.”	---
Health	---	“health wise and I feel better when I, when I walk”
Weight	---	“you look forward to the possibility of going down a size and so that, you know, that gives you that incentive to get out there and do a little more.”
Increased energy	---	“When I was walking in the afternoon I, I just felt, you know, rejuvenated”
**Best things about walking**		
Meditation time	“When I’m walking, I, I get a lot of, I can do a lot of thinking and a lot of things will come to me that I, I do a lot of studying. I can think more, it will be more clear to me and I can see a lot of things and hear a lot of things and I, like that because it, it’s nothing there to block me.”	“I like walking just cause just cause it kind of just, you can totally zone out everything. Your mind is just totally empty. You don’t have to think about anything, you just do it.”
Outside time	---	“when I walk I observe the trees, many different animals that I haven’t seen in a long time, you know, I’ll stop and look at them or, you know just take in the fresh air, you know, that can be very relaxing. That’s one of the things that I admire. Yeah, just enjoying nature and being thankful.”
Family bonding time	---	“it’s also quality time for my husband and I.”
Health benefits	---	“I know for myself with my cholesterol was elevated and I started walking. And I refused to take the medication and I just walked and so there was an improvement in my cholesterol level. So when you do exercise and you see the benefits.”
**Environmental “Convenience” factors**		
Close location	“the park just a skip and a jump from my house”	“And the next thing is I run at the park and it’s only like a two, 30 second drive away from me”
A way to measure walk	“I’m the type person needs to measure what I’ve done.”	“they have a track there now that’s really nice, you know, be able to walk on and they actually have a scale that kind of measures your distance”
**Environmental “Fun” factors**		
Aesthetics	“the scenery, especially in the spring and in the fall of the year is really beautiful and sometimes it gets your attention. You really slow down and stop and take a good look.”	“the scenery, you in the space, it’s the country that makes it fun. Just being able and, you know, hearing nature, hearing the birds and all of that stuff. It just, really does a lot for your spirit.”
People	---	“what makes it fun for me is I run into people that I know”

After discussing the best things about exercise in general, participants were prompted to discuss the best things about walking specifically. Male and female participants both mentioned meditation time as a walking benefit, “When I’m walking … I can do a lot of thinking and a lot of things will come to me that I, I do a lot of studying. I can think more, it will be more clear to me and I can see a lot of things.” Additionally, female participants noted spending time outside, family bonding time, and health benefits as additional benefits of walking. “When I walk I observe the trees, many different animals that I haven’t seen in a long time, you know, I’ll stop and look at them or, you know, just take in the fresh air, you know, that can be very relaxing.”

Participants were asked about environmental factors which make walking convenient and fun. For both males and females, having a location close to home for walking emerged as an environmental factor which would make walking easy for them. One participant said, “I run at the park and it’s only like a 30 second drive away from me.” Participants also mentioned that having a way to measure their walking distance would make walking more convenient. “They have a track there now that’s really nice, you know, be able to walk on and they actually have a scale that kind of measures your distance.” With regard to environmental factors which make walking enjoyable, male and female participants both listed aesthetics saying, “the scenery, especially in the spring and in the fall of the year is really beautiful and sometimes it gets your attention.” Female participants additionally noted that the presence of other people makes walking fun, “what makes it fun for me is I run into people that I know.”

#### Price of walking and PA

To understand the costs associated with a community program targeted at increasing walking and PA, participants were asked to identify environmental and non-environmental barriers they experience and believe others experience (see Table 4). Participants cited time, lack of motivation and tending to kids/family as non-environmental barriers to exercise. Female participants additionally added “work” as a barrier. With regard to walking specifically, female participants noted that a lack of motivation, time, and a walking partner served as barriers to walking.

**Table 4:** Summary of results (Price)

**Question & theme**	**Males**	**Females**
**Non-environmental barriers/deterrents to exercise**		
Time	“With me it’s time, you know, you’ve got to juggle a lot, you know, throughout the day”	“I think time is my worst or one of them”
Lack of motivation	“I think the things for me that make it harder is right here, just getting out there and doing it. I just will not get out there and do it like I know I should do it and like I know I have done it in the past. For me it’s just a mental thing really.”	“I have to be motivated and so I need somebody to motivate me to do it, I can’t do it by myself ‘cause I won’t, I won’t.”
Tending to kids/family	“Especially if you got kids you’ve got to deal with. So trying to fit them into the mixture of actually getting out there and doing some exercise is kind of tough.”	“taking care of your family really, you know, stops you from doing all the things that you were used to doing”
Work		“work”
**Environmental barriers/Deterrents to exercise**		
Safety	“I think part of the problem is, you know, what we mentioned earlier about safety too. Kids got the impression that sometimes it’s not safe to be out and, you know, that wasn’t a problem when I was growing up. And, you know, they, and another problem with that is, you know, as a community we have to make kids feel more safe and to be out, you know, and I think that’s part of the problem with the young people.”	“You don’t see that nowadays because the elderly is in the house kind of shielding their self from the violence and all the stuff that’s going on in the neighborhood.”
Lack of access	“where the problem comes in is those children out where they can’t come into the facilities.”	“If there’s no facility for you to exercise, you have to pay.”
Lack of adequate lighting	---	“the only thing I have a problem with if it’s dark, there’s a section that does not have any lights, so I can’t do that one.”
Dogs	---	“If I’m out and the dogs are out, I can’t go out there.”
**Non-environmental walking barriers/deterrents**		
People	“if anybody’s with me, it slows me down”	---
Lack of motivation	---	“just being motivated.”
Lack of time	---	“not having the time”
Lack of walking partner	---	“I got tired of walking by myself.”
**Environmental walking barriers/deterrents**		
Safety	“I’m not comfortable walking in certain places in certain times and I feel pretty confident I can defend, but I don’t want to be put in that position to have to defend.”	“nowadays safety has become a really big issue with walking, even in lighted areas, especially if you’re walking alone.”
Traffic	“you walk into the parking lot cars are coming, you know, so it’s just a, it’s inconvenient.”	---
Inadequate lighting	“we have to get out there early because it gets dark quicker so we don’t have a lot of light in the evenings. So if there was more lighting, you know, you could get out and walk”	“it’s just not a well lit neighborhood and when I leave from work, it’s late getting home.”
Uneven terrain	“if I step in a hole or something like that or step on something that’s not, that’s uneven that could, that could be really bad”	“the roads are hilly and then they have big rocks that hurt your feet and whatever before you get, honestly you’re tired before you reach the road, you’re tired.”
Lack of sidewalks	---	“in my neighborhood there aren’t sidewalks”
Dogs	---	“when I saw the dogs I had to minimize my walking. I mean, little dogs, any kind of dogs, you know, I’m just afraid of dogs.”
**Who influences exercising**		
Neighbors/friends	“My neighbor. Gosh, he gets out no matter what the weather’s like and he runs and I’m sure the area that, the circle that he takes is probably about a half a mile, three-quarters of a mile and he’ll run that thing and I just get tired of seeing him run it. So I had to get out there and do something myself”	“I have some ladies down from me, sometimes I will call them and let them know I’m on the way and they’ll be standing out there, you know, we walk then, down together ”
Family	“I need to do something now because of my family’s medical history.”	“I got to be able to sit here and see my children’s children, and their children”
Self	---	“it’s knowing that I have to do something, I have to be active for my own survival.”
Elderly	---	“what motivates me also too is when I see people that are older than me and they are so fit”

In an effort to more fully understand environmental barriers, participants were asked about environmental factors which make it difficult to exercise and walk. Previously mentioned as an important factor to be included in a walking trail, safety emerged a major environmental barrier for participants. Lack of access to facilities in the community was also a theme that emerged as an environmental exercise barrier, “If there’s no facility for you to exercise, you have to pay.” Female participants also listed inadequate lighting and dogs as environmental barriers to both exercise and walking. Specific to walking, female participants noted that uneven terrain interfered with their walking, “the roads are hilly and then they have big rocks that hurt your feet, honestly you’re tired before you reach the road, you’re tired.” Male participants also mentioned traffic. To overcome these barriers, participants suggested increased lighting as a solution, “come on through and put in more lighting in our neighborhood.”

With regard to interpersonal influences on walking and exercise behaviour, male and female participants cited neighbors, friends, and family members as some of the most important influences. For example, “My neighbour, gosh, he gets out no matter what the weather’s like and he runs and I’m sure the area that, the circle that he takes is probably about a half a mile, three-quarters of a mile and he’ll run that thing…” Female participants also thought they, themselves, as well as the elderly influence their walking and exercise behavior and said the following, “it’s knowing that I have to do something, I have to be active for my own survival” and “what motivates me also too is when I see people that are older than me and they are so fit.”

#### Promotion of walking and PA

To determine how participants would promote a walking trail, participants were asked what they would say and do to get others active (see Table 5). Additionally, they were asked who they would get out walking first and what messages would be best to use to get others out. Both male and female participants shared that walking with others would be a primary strategy for getting others motivated to walk, and that they would discuss health and inspiration with others. Female participants additionally cited the use of incentives, competition, and the provision of childcare as effective strategies while male participants suggested the use of role models and partnering with organizations to increase motivation for walking.

**Table 5:** Summary of results (Promotion)

**Question & theme**	**Males**	**Females**
**Overcoming environmental barriers/deterrents to exercise**		
Increase lighting	“come in through and put in more lighting in our neighborhood.”	---
Exercise at home	---	“I would say get started in your house, you know, in your own family, you could put it and stay in your house.”
Time management	---	“I say time management. Cut back on watching TV”
Motivation/determination	---	“you just have to put your mind to it, get out there.”
Help from others	---	“I need somebody to help me be there when I’m supposed to”
**Overcoming barriers to walking**		
Access to walking place	“I think one thing that would enhance walking and help in general just having just a universal complex that had walking trails inside and out”	“if we get the track, a walking track or something in the area”
Motivation/discipline	---	“You say well everyday I’m gonna do my best to walk at a certain time and you have to discipline yourself like you discipline yourself to get up every morning and go to work.”
Education/discussion	---	“I believe education is power and I think educating people on the importance of the benefits of exercise and how we can eliminate a lot of the … heart attacks, strokes”
Walk with others	---	“If I have somebody that I know that’s going to do it with me, it’s not problem.”
Use alternatives	---	“you can walk in place at your home”
**Do to get others active**		
Walk with others	“And like you were just saying networking as you walk.”	“and it does make a difference when you have someone that’s right there to stay on you every day, you know, let’s do it, let’s not give up, you know. So I think motivating groups, I think walking groups, or whatever the case may be. Just banding together with people that have things in common towork together and make some things happen.”
Motivation	“it gonna have to be a community effort and it’s still going to boil down to the individual, even doing the community effort.”	---
Role modeling	“I think if, if we as adults model the fact that we need to be out walking and our children see it and our children pick up on it, and they start doing it, it becomes a habit or, or a, a routine for them.”	---
Incentives	“In the long run it saves everybody money in the community. Yep, if they could motivate us by giving discounts.”	“think that maybe sometimes we have to throw out some incentives to get folks fired up”
Partner with organizations	“But, you know, I was curious, but then I think that’s part of the, I reckon either county council or city, whoever own the park, need to do a better job of informing people of places like that so, you know, we can realize it’s there.”	---
Competition	---	“something like the ‘biggest loser”
Provide childcare	---	“Provide a babysitting service.”
Provide transportation	---	“transportation, we’d provide transportation.”
**Say to get others active**		
Health information	“exercise brings on life, non-exercise might bring on death. [Laughter] You know what I’m saying? We’ve got to get that instilled in them.”	“Just like they advertise that smoking is bad and it causes cancer, tell them what not walking causes.”
Inspirational message	---	“…it’s about purpose.”
**Who in neighborhood would encourage to get walking first**		
Community snowball effect	“Once somebody starts, especially if you start with your immediate family, and it, it takes like one family to really get involved in it, you know, in the, in the neighborhood. And then the other children get involved and the other parents because I was telling them last night even at the meeting, we’ve got to go back into our communities.”	“One house to the next. You just start that fire… one house to the next.”
Younger people	“to get this back right, to get us in good health again you don’t have to start, not with old rascals like me, it’s the young people, it’s the young people.”	“I see in our neighborhood a tendency to target the younger people because they spend a lot of time inside now and, on the computer, on their cell phones, you know, that kind of thing. So we see a lot of our younger people becoming obese and, you know, you hear the commercial on the radio in the morning that, you know, we might outlive our children, you know, and that’s sad.”
Spouse	---	“my husband.”
**Best way to communicate messages**		
Word of mouth	“word of mouth.”	“And that person will affect two people and then so on and so on. And you just keep going and there’s that word of mouth.”
Church	“Well a, a lot of times with churches, you have people in the church who, who are knowledgeable. And, and a lot of times you go to church there, there are health care providers. There are lots of people with talent in there that attend church and everybody knows and they would appreciate or, or they would give that time and that attention to you.”	“churches, faith-based communities could be an avenue”
Local organizations/groups	“neighborhood groups”	---
Sporting events	“some type of announcement at a sporting event because a lot of people like a sporting event.”	“For example, you have someone to go out to the ball field, to the I mean, at school or actually on site to speak to children about health”
School	---	“the school is a very important avenue too that we have to consider.”
Promotional events	---	“having street cookouts like, you know, the block cookouts”
Flyers	---	“Flyers, you would need flyers in the country, something to get through to that particular house.”

Indicating the importance of getting certain groups out to walk, both male and female participants said that younger people should be a target, and should get out walking first. Participants also agreed that a community snowball effect would get a variety of people out, “One house to the next. You just start that fire… one house to the next.”

Participants were able to come up with a variety of methods to spread the word about a walking trail, with word of mouth communication via “churches, faith-based communities” as the optimal way to communicate messages. Female participants additionally liked the idea of using flyers and communicating through schools while male participants suggested partnering with local organizations and neighborhood groups.

#### Social marketing campaign development process

Focus group data were used to develop the intervention community social marketing campaign. Steering committee members including a community liaison, program coordinator, walking leaders, city leaders, community residents, a local pastor, city police and a communications firm worked to develop a comprehensive social marketing campaign (Tables 6 and 7). Strategic planning focused on themes around motivators for walking and overcoming barriers to walking. From these data and ongoing community input an over-arching social marketing goal was developed to motivate citizens to use the neighbhorhood walking path with others regularly and safely for increasing PA and improving health of mind, body, soul and community. Five specific objectives were developed which included (1) Increasing perceptions of the safety of walking on the path with others, (2) Creating the attitude that walking on the path will improve physical health, (3) Creating attitudes that walking on the path will improve mental health and well-being, (4) Increasing confidence in walking on the path regularly, and (5) Increasing feelings of social connectedness and perceptions of community pride by walking on the path with others.

**Table 6:** Process of developing social marketing promotion strategies

**Focus group themes →**	**Objectives →**	**Promotion**
• Having access to nearby, safe places to walk promotes activity	• Walking benefits physical health	• *Calendar*- included photographs of community walkers, objectives and goal-setting exercises
• Walking increases energy, benefits health, and provides stress relief and opportunities for meditation and spirituality	• Walking benefits mental and spiritual health	• *Door Hangers*- invited new walkers, reinforced objectives, and highlighted program incentives
• Word-of-mouth among neighbors, family, and friends is the most influential process for affecting community behaviors	• Walking builds your confidence for being active	• *Peer Walking Leaders*- facilitated grassroots networking, reinforced objectives
	• Walking builds social and community connections	
	• Walking is safe	

**Table 7:** Social marketing components and elements

**Social marketing components**	**Theory elements**	**Practice elements**
Product	Desired behavior targeted by SM.	• Increased walking generally
		• Participating in the PATH walks
Price	The social, economic, and psychological and physical costs involved in achieving the product.	• Devoting time to walking; sacrificing time typically spent on other activities
		• Driving or securing transportation to the PATH walks
		• Expending mental and physical energy
		• Perceived threats of injury and to personal safety
Place	The setting, community context, or distribution channels for the product.	• Community centers
		• Churches
		• Neighborhood walking path
Promotion	All actions designed to make the audience aware of the product and its benefits.	• Distributing campaign themes through calendars, door hangers, and during walks
		• Grassroots strategies, Pride Strides
		• Local media involvement, newspaper articles
Positioning	Framing the product so that the perceived benefits are maximized and the perceived costs are minimized.	• Distributing messages that brief walks positively affect health
		• Providing injury prevention exercises and information
		• Ensuring that off-duty police are available to support the program

The community steering committee also closely guided the development of a program name, logo, tag line, and the overall social marketing approach. Information from the focus groups was used to guide the development of promotional benefits of walking specifically. These themes were used by the steering committee to develop the messages on the calendars and door hangers (e.g., improve physical and mental health, social connectedness etc.). The community was concerned that the marketing materials be functional and attractive to their residents. As such, the communications firm provided the steering committee with several mock program names and logos from which the community could choose the one that best captured their vision. During this process, the steering committee developed the social marketing tag line, “A healthy you and a healthy me makes a healthy community.” The steering committee and the communications firm agreed that while print materials are necessary to increase awareness of the program, a grassroots approach prioritizing personal connection with individuals would be the most effective strategy for achieving the product of walking behavior change. With these considerations in mind, a three-pronged approach was developed for promotion of the social marketing campaign, and the approach was based on the themes obtained through community focus groups and tailored for delivery of the five objectives (Table 6). The primary means for delivering messages related to the five objectives was a 12-month calendar that was given to community “first time” walkers who participated in a PATH walk. Walking leaders also made a point to deliver calendars to neighbors in the target community by going door-to-door. The calendar was designed to highlight the local community through photographs of residents walking on the path. Each month the focus on one of the five objectives was highlighted in the calendar, along with goal setting and tracking of walking progress. Thus, the calendar not only served as a functional tool for community residents, it was also a unique tool for increasing self-efficacy for walking.

The second set of materials included door-hangers that could be attached to neighbors front doors and that were designed to increase opportunities for personally inviting new walkers to join a PATH to HEALTH walk. Door hangers were distributed throughout the target community by PATH participating walkers and by walking leaders. The door hangers aimed to serve three purposes. First, they were used to personally invite new walkers to the group, facilitating interpersonal interaction and verbal communication about the program. Second, they complemented the calendar by reinforcing the monthly objectives and messages. Finally, they highlighted the incentives program, which would encourage walkers to do at least five walks per month to receive prizes related to the month’s theme such as hand-held fans and water bottles.

The third key element of the social marketing campaign targeted the involvement of peer walking leaders. Through grassroots networking, the program engaged residents to lead peer walking groups called Pride Strides. Peer leaders would organize and lead walks for their families, friends, and professional organizations, not only by utilizing the calendars and door hangers, but also by using a field guide that outlined project details and reinforced the messages from the calendars and door hangers with talking points, inspirational poems and prayers that reflected the social marketing objectives. Thus, the peer leaders further facilitated an interpersonal channel of communication for reinforcing the social marketing messages.

Finalization of the social marketing plan centered on developing the messages, designing the print materials, and developing a protocol for implementation. In order to maximize the relevance and effectiveness of the messages, the social marketing firm encouraged the community steering committee to develop the messages that would be used throughout the calendar, door hangers, and field guide for Pride Stride leaders. Likewise, the community felt it was important that the calendar include photos of actual residents on the walking path, so the communications firm conducted a photo shoot with community partners and residents. Finally, a universal protocol for distributing social marketing materials that focused on behavior modification theory was developed to ensure the social marketing campaign was implemented with fidelity.

## Discussion

This study highlights the importance of using a collaborative community process to develop a social marketing campaign to promote walking in underserved neighbourhoods and communities. Focus group data helped us to develop program deliverables that focused on a grassroots approach to inviting local neighbors out to walk. Door hangers and calendars that were culturally tailored were distributed to promote important themes for improving safety and access for walking as well as the benefits of improving health and being more socially connected. It has been noted that involving community input directly in the social marketing process increases the integration of a program into established community structures, which may make the program more effective and sustainable [44]. A collaborative approach to social marketing included engaging active participants in the process of program development, and working with community groups in designing, implementing, and evaluating the social marketing program prospectively [45].

One of the major strengths of this study was the use of community participation to ensure the social marketing approach was tailored and truly fit the needs of the intervention community. Residents from the PATH full intervention community and steering committee members from this community continually provided feedback on social marketing objectives, messages and modes of delivery. Consistent with our approach, Farmer and colleagues [46] have highlighted the important role of community involvement for increasing the probability of a social marketing program’s success and its role in building community capacity. In addition, they suggest that involving the community at all stages or components of the social marketing process (i.e., in the “5 P’s”) may result in a program that is purposefully tailored to the needs of the individual community, and therefore more likely to result in intended effects [46]. In this study both community focus groups and steering committee input were key in developing a tailored social marketing strategy to promote walking on the neighbourhood trail. Future studies should also distinguish between whether trail use may be a function of walking for leisure or for transportation [47].

Formative focus groups conducted in the PATH intervention community, as well as efforts to obtain ongoing input from the community steering committee, led to the more specific tailoring of the social marketing campaign around the community’s concern for safety and the desire for a grassroots approach. A limitation of the formative process, however, was that only several of the focus groups were done with the target community neighbourhood association. Given that the PATH trial was a randomized trial several of the focus groups that were done at a near by school in a community matched on geographical demographics were conducted in advance of randomization to reduce the amount of time between program planning and implementation. However, a related strength of this study is the use of tailored messages that matched the needs of the target community. Previous research conducted during the development of the PATH trial highlighted the importance of safety issues for residents living in disadvantaged conditions [34]. Residents in the PATH full intervention community described concerns and provided specific suggestions on how to overcome barriers related to criminal activity, such as street gangs, in addition to concerns regarding stray dogs, lighting, and traffic.

One of the most important features of the PATH social marketing campaign was the grassroots approach to delivering messages to African American community members. Traditionally, campaigns designed to reach broad-based audiences have used mass media-based strategies to communicate program messages [18,48]. For example, Beaudoin and colleagues [49] used a mass media campaign to increase walking in a low-income, African-American urban population in New Orleans. Messages were promoted through television advertisements, radio advertisements, taillight bus signs, large side-panel bus signs, taillight streetcar signs, and large side-panel streetcar signs. Similarly, some of the strategies used in the VERB campaign to increase PA in ethnically diverse youth and their parents included advertising on television, in print, on billboards, through a website, and through school and community-based promotions [50]. Unlike these studies, the PATH social marketing campaign used interpersonal channels of communication. Program deliverables (e.g., the 12-month calendar featuring local leaders and residents, door hangers) were designed to foster communication and were meant to be spread by local, familiar faces in the community. In addition, media efforts like local newspaper testimonials were used to highlight the community connections and safe opportunities for walking. A review of population-based interventions targeting active living in ethnic minority communities showed that in addition to non-interpersonal channels for message delivery, studies that specifically included ethnic minorities place greater emphasis on factors such as building coalitions, mobilizing social networks, and tailoring culturally specific messages [29]. In this study, interactions and conversations between neighbors, family members and friends were at the core of the grassroots approach to social marketing.

Not only were interactions between neighbors, family members, and friends important as an approach to delivering messages, the community also identified them as essential targets for message content. One of the five social marketing objectives was to increase feelings of social connectedness and perceptions of community pride by walking on the path. In addition, the social marketing tag line, “A healthy you and a healthy me makes a healthy community” highlighted the community’s emphasis on the relevance of appealing to community connectedness. Consistent with Yancey and colleagues [29], this study showed the importance of incorporating community norms and activities in developing a social marketing campaign to promote walking in underserved communities. The PATH social marketing campaign was novel in that it targeted community norms by focusing specifically on family and community connectedness in both the overall approach and the tailored messages.

## Conclusions

Overall, this study suggests that developing and implementing a social marketing intervention for promoting walking in an underserved community requires ongoing community involvement. The final tailoring of the social marketing program in the target full intervention community was key to developing a program that was accepted and liked by community residents. The formative process described in this article is a model that could be used to develop community-based social marketing interventions in other underserved communities to promote walking and PA. Specifically, focus group data and community steering group involvement was critical for developing a grass roots social marketing strategy to improve safety and access for walking that is especially important for low income, ethnic minority communities.

## Competing interests

The authors declare that they have no competing interests.

## Authors’ contributions

DKW is the Principal Investigator on the project and provided oversight for the entire project. She was the major contributor of data collection, interpretation of the data analyses and to writing of the article. SMS is a doctoral student who served as the intervention coordinator on the project. She contributed to data collection, the interpretation of the data analyses and to writing of the article. NNT is a doctoral student who served as the project director on the project. She contributed to writing the article and to the data collection and interpretation of the data analyses. SMC is a doctoral student who served as an intervention process evaluation coordinator. She contributed to the writing of the article and to the interpretation of the data analyses. SFG is a Co-Investigator on the project. She contributed to the writing of the article and to the data collection and interpretation of the data analyses. AW is a Co-Investigator on the project who contributed to the study design of the qualitative study and to the data interpretation and writing of the article. MF is a Co-Investigator on the project who contributed to the study design of the qualitative study and to the data interpretation and writing of the article. BG is are community partner who assisted with recruitment, data collection, and contributed to the writing of the article. PVB is a graduate student who assisted with conducting the focus groups, data collection, interpretation of the data analyses and to the writing of the article. All authors read and approved the final manuscript.
